# Evaluating the Response Time of an Optical Gas Sensor Based on Gasochromic Nanostructures

**DOI:** 10.3390/s21248472

**Published:** 2021-12-19

**Authors:** Igor A. Nechepurenko, Daria P. Kulikova, Vladimir V. Kornienko, Konstantin N. Afanasiev, Landzhik A. Shekoyan, Alexander V. Baryshev, Alexander V. Dorofeenko

**Affiliations:** 1 Dukhov Research Institute of Automatics (VNIIA), 22 Suschevskaya, 127055 Moscow, Russia; nechepurenko@phystech.edu (I.A.N.); dp.kulikova@yandex.ru (D.P.K.); vladimirvkornienko@yandex.ru (V.V.K.); kavacuum@mail.ru (K.N.A.); baryshev@vniia.ru (A.V.B.); 2 Department of Theoretical Physics, Moscow Institute of Physics and Technology, 9 Institutskiy Pereulok, 141700 Dolgoprudny, Russia; 3 Faculty of Physics, M.V. Lomonosov Moscow State University, 1 Leninskie Gory, 119991 Moscow, Russia; 4 Institute for Theoretical and Applied Electromagnetics RAS, 13 Izhorskaya, 125412 Moscow, Russia; 5 Department of Theoretical Physics, Adyghe State University, 208 Pervomayskaya, 385000 Maykop, Russia; lanjsn@yandex.ru

**Keywords:** hydrogen sensors, optical sensors, gasochromic oxides, tungsten trioxide, Kretschmann scheme

## Abstract

We propose a method for determining complex dielectric permittivity dynamics in the gasochromic oxides in the course of their interaction with a gas as well as for estimating the diffusion coefficient into a gasochromic oxide layer. The method is based on analysis of a time evolution of reflection spectra measured in the Kretschmann configuration. The method is demonstrated with a hydrogen-sensitive trilayer including an Au plasmonic film, WO_3_ gasochromic oxide layer, and Pt catalyst. Angular dependences of the reflectance as well as transmission spectra of the trilayer were measured in series at a constant flow of gas mixtures with hydrogen concentrations in a range of 0–0.36%, and a detection limit below 40 ppm (0.004%) of H_2_ was demonstrated. Response times to hydrogen were found in different ways. We show that the dielectric permittivity dynamics of WO_3_ must be retrieved in order to correctly evaluate the response time, whereas a direct evaluation from intensity changes for chosen wavelengths may have a high discrepancy. The proposed method gives insight into the optical properties dynamics for sensing elements based on gasochromic nanostructures.

## 1. Introduction

Gas sensors are widely used in industry, environmental monitoring, and other fields [1]. Most common are resistive gas sensors based on thin films of metal oxides [2,3,4,5], and they are manufactured commercially [6]. Despite intensive development, they are far from being ideal, particularly, these sensors are not fully selective [7], and they have working temperatures from 100 to 400 °C. To avoid heating, optical sensors based on gasochromic properties of metal oxides (WO_3_, NiO, etc.) paired with catalyst (Pd, Pt) [8,9,10,11] and on the solubility of gas in metals (Pd, Pt, Mg, etc.) [12,13,14,15] are elaborated. These sensors use the property of materials to change the extinction coefficient and/or refractive index as a result of reaction with a gas [3]. Optical gas sensors of H_2_, CO, and other gases demonstrate a detection limit of tens and hundreds of ppm [16], which is enough for most applications. An important issue—protecting the sensing materials from degradation by using polymer coatings—is under investigation [10].

Hydrogen detection has become a very important task in the view of increasing demand for alternative energy solutions. Tungsten trioxide has a pronounced response to hydrogen, which made it one of the most promising platforms for optical H_2_ detection [11,17,18,19,20,21]. Tungsten trioxide is characterized by two response mechanisms [22,23]: (i) the formation of tungsten bronze (HWO_3_) is typical of a polycrystalline oxide film, and (ii) the appearance of oxygen vacancies (WO_3–*x*_) is discussed for porous amorphous WO_3_ films. These mechanisms are governed by specific chemical reactions determining the response of tungsten trioxide to hydrogen [24,25,26,27].

Optical properties of pristine (uncolored) tungsten trioxide are well studied [17,18]. In the *gasochromically* colored state, the refractive index of bulk WO_3_ was retrieved from transmittance [19], whereas more accurate measurements with spectroscopic ellipsometry were demonstrated for the *electrochromically* colored state [24,28,29]. Particularly, the optical constants at an electrochromic response of WO_3_ are reported [24]. It was shown in Ref. [30] that the optical properties of tungsten trioxide allow obtaining information on the material structure such as porosity (fraction of voids) and fractions of polycrystalline and amorphous phases. It is worth outlining that most works discuss the optical properties of the pristine and completely colored states of WO_3_ or only provide data on the transmittance dynamics [31,32,33]. Thus, there is a lack of data from in situ measurement of complex dielectric permittivity *ε* ≡ *ε*′ + *i ε*″ in the partially colored state, which is necessary for understanding material response and designing gas sensors.

To obtain these data, we consider the Kretschmann configuration as a useful optical scheme [26,34,35], which has proven its reliability and sensitivity. In the Kretschmann configuration, the dispersion characteristics of gasochromic oxide can be restored. For example, the refractive index of WO_3_ films of various thicknesses was retrieved using the plasmon resonance in a range of 405–633 nm [36]. The same is done for noble metal films [36], demonstrating that the results of reconstructing the optical constants for the data obtained in the Kretschmann scheme and using spectral ellipsometry are in good agreement.

In the present paper, we investigated dynamics of optical response of Au/WO_3_/Pt trilayers in the H_2_-containing atmosphere by utilizing different optical geometries and determined the detection limit not exceeding 40 ppm. In these experiments, we retrieved the time-dependent dielectric permittivity at a single wavelength. Our analysis showed that dynamic characteristics such as the response time should be determined from the material permittivity, not directly from transmittance or reflectance. This result is important for both fundamental understanding of the material response and correct processing of data retrieved from an optical sensor.

## 2. Experimental and Calculation Details

**Figure 1:**
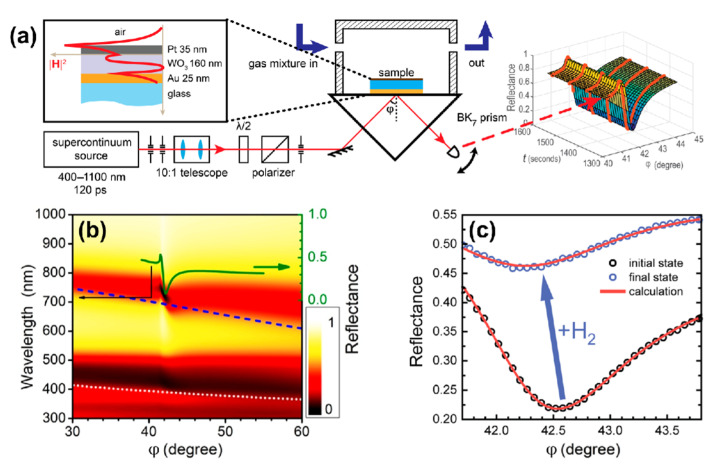
(**a**) Kretschmann scheme for measuring the optical response of tungsten trioxide to hydrogen. The inset shows the structure of the trilayer under study as well as the distribution of the magnetic field intensity in the presence of the *p*-polarized excitation wave at a wavelength of 705 nm. (**b**) Calculated angular and spectral map of reflectance for TM- (*p*-) polarized wave; additionally, a reflectance curve at a wavelength of 705 nm is shown (solid green line). Dispersion curves for the first (dashed blue line) and the second (dotted light gray line) waveguide modes were calculated for the case of thick (200 nm) metal layers. (**c**) Change in the angular dependence of reflectance under exposure to 0.36% (3600 ppm) of hydrogen.

The Au/WO_3_/Pt layers comprising the sensing element were deposited by electron beam evaporation onto a glass substrate (DeltaLAB, Spain) (Figure 1а). The pelletized WO_3_ targets were heated by means of an electron beam collimated from the DC-heated tungsten filament cathode. Layers’ thicknesses were controlled by optical transmittance of a reference sample (for Au and Pt evaporation) and by an interference control (for WO_3_) at 630 nm. The substrates were pre-rinsed with isopropyl alcohol and cleaned in a gas discharge at 10^–2^ Tor. During evaporation, the chamber was evacuated to a base pressure of 10^−5^ Pa, and an accelerating voltage of about 7.5 kV was used. The substrate was kept at room temperature.

To study the sensing element response dynamics, an experimental setup was assembled (Figure 1а). We used a pulsed supercontinuum light source (Fianium, NKT Photonics) in combination with an acousto-optical filter yielding a mean power of 1 mW for a 4 nm FWHM beam in a range of 400–1100 nm. Reflected light power versus time was measured with a silicon photodetector. The same dynamics was measured for transmitted light at normal incidence.

The optical thickness of the WO_3_ slab in the trilayer was chosen to be approximately equal to half of an operating wavelength of 705 nm at normal incidence. Figure 1b shows the calculated dependence of reflectance on an angle of incidence (*φ*) and wavelength (*λ*) for the *p*-polarized light; in this calculation, dielectric permittivities of ${\mathrm{WO}}_{3}$, Pd, and Au were taken from Refs. [29,31,37], respectively. One can see that the structure supports both the *surface plasmon polariton* (SPP) on the gold film and a *quasi-guided mode* (QGM) in the WO_3_ layer. Dispersions of *guided modes in case of* the WO_3_ layer on a thick (>200 nm) Au layer are shown with the dashed (first mode) and dotted (second mode) curves. A disturbance of the first branch at λ ≈ 750 nm was due to the interaction between QGM and SPP.

## 3. Sensing Element Response in Case of Monochromatic Light

**Figure 2:**
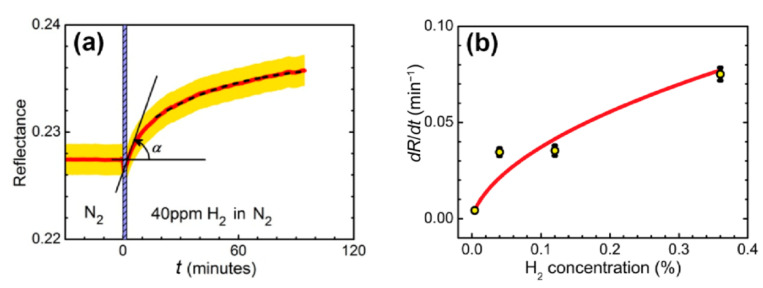
(**а**) Reflectance dynamics of sensing element exposed to 40 ppm hydrogen in nitrogen. The measurements were performed at the wavelength of 705 nm and angle of incidence of 42.5°. Red line—experimental data, yellow stripe—estimated measurement error. The dashed line is an exponential fit. Hydrogen is supplied at *t* = 0 min, the vertical shaded stripe width shows the stabilization time of the gas stand. (**b**) Dependence of the response of the WO_3_-based sensing element on the hydrogen concentration. Hydrogen concentration: 0.004−0.36%, carrier gas: nitrogen. The line shows the approximation based on the response model with the formation of tungsten bronzes in accordance with expression (1) from [38].

In the experiment, the sensing element was placed into a metallic cuvette with a free volume of 1.5 mL and was subjected to a constant flow 100 mL/min without overpressure of gas mixture. The experiment was carried out at room temperature. The cuvette containing trilayer was fed with gas mixtures as follows: (i) oxygen 21% in nitrogen for 60 min to ensure identical starting conditions, then (ii) hydrogen in nitrogen for 180 min. We used dry gas mixtures with the relative humidity of <1%. In the nitrogen–hydrogen atmosphere, a drastic change in the *R*(*φ*) curves was detected at a high concentration of H_2_ (Figure 1c) and was easily resolved for a concentration of 0.004% (40 ppm), see Figure 2a, which gives the upper estimate of the detection limit.

For applications, a fast analysis of the sensor response is necessary. Although the overall evolution of the reflectance may take tens of minutes, the presence of hydrogen can be detected within the first tens of seconds after the reflectance starts to change. For this purpose, the rate of change of the reflectance in the initial moment of time, dR/dt, may be considered as a sensor response (Figure 2а). The dependence of dR/dt on hydrogen concentration was determined for a sensing element placed in a gas cuvette and exposed to four different hydrogen concentrations (Figure 2b).

## 4. Influence of Spectral Features on Sensing Element Response Times

**Figure 3:**
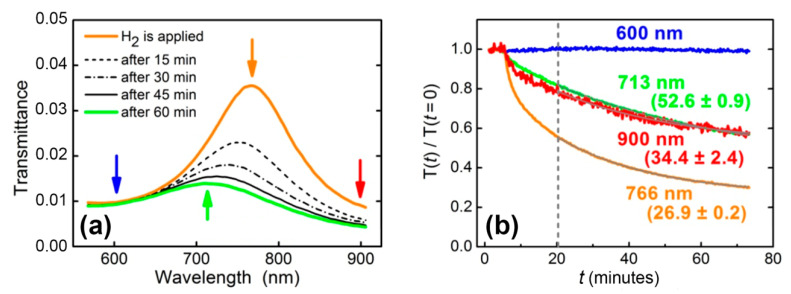
(**а**) Transmittance dynamics of WO_3_-based sensing element exposed to 200 ppm hydrogen in nitrogen. (**b**) Normalized transmittance for selected wavelengths (shown in Figure 3а by arrows): initial position of the maximum (766 nm); the position of the maximum 1 h after the supply of hydrogen (713 nm); left (600 nm) and right (900 nm) boundaries of the observation area. Response times retrieved from a single-exponential fit ($t_{1}$) are shown near the curves (in minutes).

At the second stage, the same Au/WO_3_/Pt trilayer was studied in the geometry of normal incidence, and spectral characteristics instead of angular ones were retrieved. Transmittance was measured periodically in time during exposure to 200 ppm hydrogen in N_2_ (Figure 3a). The observed feature at 766 nm was due to the interference maximum for the 160 nm-thick WO_3_ layer. In processing these data, a naive approach is fitting transmittance data with the exponential function, *T*(*t*) = *T*(0) exp(−(*t* − *t*_0_)/*t*_1_) (dashed line in Figure 3b), at long enough times ($t>t_{0}$, $t_{0}=15$ minutes after the start of the gas flow—Figure 3b). This fit gives *t*_1_, which is the response time of the structure. The major failure of this approach is that *t*_1_ turns out to be dependent on the wavelength, i.e., *t*_1(_λ*_i_*) ≠ *t*_1(_λ*_j_*). Thus, the direct exponential fit of the transmittance dynamics does not give a universal response time characterizing the change in material properties (dielectric permittivity).

The difference between response times arose due to the pronounced resonant features in the transmittance of the structure under study (Figure 3а). This argument was confirmed by a numerical calculation of transmittance through the same Au/WO_3_/Pt trilayer. The dielectric permittivity of the WO_3_ layer was modeled as a sum of the slow-varying term (*ε* = *n*^2^ + 0 *i* = (A + B/*λ*^2^)) and the resonance response in the form:

(1) $$\varepsilon_{TOTAL}={\left(A+\frac{B}{\lambda^{2}}\right)}^{2}+\frac{f{\left(1/\lambda_{0}\right)}^{2}}{{\left(1/\lambda_{0}\right)}^{2}-{\left(1/\lambda \right)}^{2}+i\Gamma /\lambda_{0}}.$$

**Figure 4:**
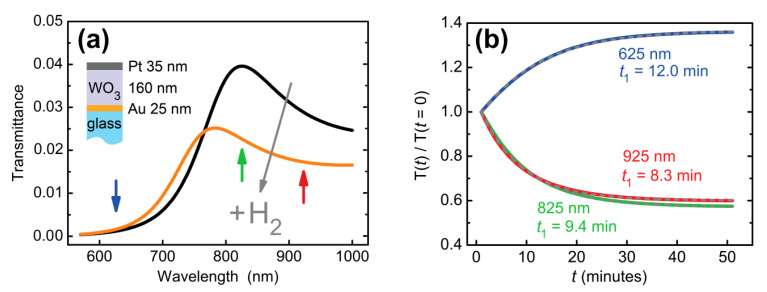
Calculated transmission dynamics of an Au (25 nm)/WO_3_(160 nm)/Pt (35 nm) trilayer. (**a**) Transmittance before and after hydrogen supply. (**b**) Transmittance at a given wavelength as a function of time. Refractive index changes of Pt were neglected for the purpose of illustration of response time variations due to two resonance in a dielectric WO_3_ layer. Exponential decay times (*t*_1_) at different wavelengths are indicated next to the curves.

Here, the first term corresponds to the bleached (non-colored) state of WO_3_ with the following parameters’ values: *A* = (1.9227 ± 0.0009) and *B* = (0.03064 ± 0.0006) μm^2^. The second term describes the response of WO_3_ to hydrogen. The central wavelength, *λ*_0_ = 1.1 μm, corresponds to the position of the gasochromic absorption peak of WO_3_ [39]. The transmittance spectral curves were calculated for different oscillator strengths. We took the oscillator strength *f* exponentially changing in time, *f* = *f*_0_ ∙ (1–exp(−*t*/*t*_1_)), with the amplitude *f*_0_ = 0.1 chosen to provide the same difference between initial and final states as had been observed in the experiment (see e.g., Figure 3a) and response time *t*_1_ = 10 min. Significant deformation of the calculated transmittance curve was observed (Figure 4a). Response times were calculated for different wavelengths. They appeared significantly (>20%) different: *t*_1_ (625 nm) = 12.0 min, *t*_1_ (825 nm) = 9.4 min, *t*_1_ (925 nm) = 8.3 min. This result is consistent with the experimental data—see Figure 3b. The reason for such a difference in response times is the presence of the transmittance resonance, which shifts as the refractive index changes. In general, the response time depends both on the magnitude of the spectral shift, on the quality factor of the optical resonance, and on the selected working wavelength.

Thus, for the resonant structures, which are widely used in optical gas sensors, direct determination of the material response time via the transmission spectra is not valid. Even in the case of the refractive index of sensing material changing homogeneously in the full spectral range, the transmittance response times differ at different wavelengths. Particularly, in the aforementioned example, relaxation times measured at the wavelengths of 713, 766, and 900 nm were 52.6 ± 0.9, 26.9 ± 0.2, and 34.4 ± 2.4 min, respectively. An accurate dynamics description requires the retrieval of time-dependent optical constants (complex refractive index or dielectric permittivity) of gasochromic materials. Below, using the WO_3_-based sensing element, we demonstrate how the time dependence of the dielectric permittivity can be calculated.

## 5. Time-Dependent Refractive Index of Tungsten Trioxide Interacting with Hydrogen

The complex refractive index of WO_3_ and its dynamics were defined in two experiments. First, the spectral ellipsometry allowed us to find the dielectric permittivities and layer thicknesses in the trilayer: gold—(25 ± 10) nm, tungsten trioxide—(160 ± 10) nm, platinum—(35 ± 10) nm. When using these parameters, the agreement between calculated and measured reflectance was rather precise (Figure 1с). Theoretical (red lines) and experimental (circles) reflectance curves are shown in the absence (black) and in the presence (blue) of 3600 ppm hydrogen. This high concentration was necessary for the reliable retrieval of time-dependent permittivity.

**Figure 5:**
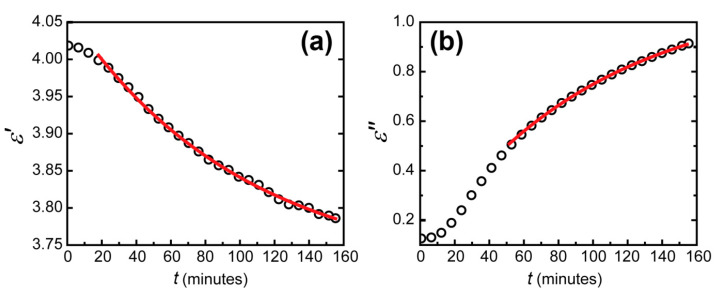
Dependence of the imaginary (**a**) and real (**b**) parts of the dielectric permittivity of tungsten trioxide on time (black circles) for an excitation wavelength of 705 nm and the exponential fit of this dependence on time (red lines).

Second, reflectance measured in series in the Kretschmann geometry allowed a real-time dielectric permittivity tracking. The simultaneous fit was achieved for all the reflection contours by searching for parameters of the WO_3_ layer. It is important to note that the resonant behavior of the contours imposes strict limits on the possible parameters of materials composing the sensing element, including layers’ thicknesses. These parameters except for the WO_3_ complex refractive index were considered constant. To calculate the parameters of Pt and Au, two contours were fitted. The first one was measured a few minutes after the trilayer exposure to hydrogen, and the second one was measured when reaching the equilibrium condition after three hours. As a result, the thicknesses and complex refractive indexes of all the layers were calculated. With these parameters, we then fit transient spectra using the refractive index of the WO_3_ layer as a fitting parameter. Resulting real and imaginary parts of WO_3_ permittivity dependent on time are shown in Figure 5. Then, an exponential fit was applied to determine the actual material response time.

The errors of fitting the real and imaginary parts of dielectric permittivity of WO_3_ appeared to differ significantly. The determination coefficient reached 0.9999 for the imaginary part and 0.995 for the real part. This difference can be seen visually (see Figure 5). It was due to the different precision of dielectric permittivity parts determination. From the data obtained in our experiments, the imaginary part was determined more precisely than the real part for two reasons. The first one was the larger change in the imaginary part of WO_3_ permittivity than of the real part. This imaginary part increase led to additional absorption in the WO_3_ layer, which led to its coloration. The second reason was the physical nature of the imaginary and real parts of the dielectric permittivity. The real part is responsible for the optical thickness of the WO_3_ layer, while the imaginary part determines the absorption coefficient. In the Kretschmann configuration, due to zero transmission, the total reflectance depends solely on the optical absorption. The absorption without hydrogen occurs generally in the metal layers. This absorption was well determined when fitting the initial state of the WO_3_-based sensing element. Thus, when hydrogen was applied, an additional large absorption in WO_3_ was calculated with very good precision. In contrast, an additional phase for the electromagnetic wave in the WO_3_ layer occurs at any hydrogen concentration.

For further understanding the interaction of hydrogen with the WO_3_ layer, we developed a theoretical model based on the diffusion of color centers in the WO_3_ layer along the *x*-axis across the layer. This model allows us to find the asymptotic behavior of the dielectric permittivity of WO_3_ after continuous exposure to hydrogen. Let us consider the diffusion equation in a film with an impenetrable boundary on the one side, with a constant concentration on the other side, and zero initial concentration:

(2) $$\begin{array}{l} \frac{\partial n\left(x,t\right)}{\partial t}=D\frac{\partial^{2}n\left(x,t\right)}{\partial x^{2}},\\ n\left(l,t\right)=n_{0},\;\;\;{\frac{\partial n\left(x,t\right)}{\partial t}|}_{x=0}=0. \end{array}$$

Here, D is the diffusion coefficient, l is the layer thickness, and $n_{0}$ is the constant concentration of hydrogen atoms at the boundary. The solution to the diffusion equation with such boundary conditions can be found in the form of a series:

(3) $$n\left(x,t\right)=n_{0}\left(1-\frac{4}{\pi }\right)\sum_{i=0}^{\infty }{\left(-1\right)}^{i}\frac{\cos\left(\frac{\pi (2i+1)x}{l}\right)\exp\left(-\frac{t\pi^{2}D{\left(2i+1\right)}^{2}}{4l^{2}}\right)}{2i+1}.$$

A feature of this solution is a fast convergence at long times t. At long times, the concentration in the layer is $n\left(x,t\right)\approx n_{0}\left(1-\frac{4}{\pi }\right)\cos\left(\frac{2\pi x}{l}\right)\exp\left(-\frac{t\pi^{2}D}{4l^{2}}\right)$. The optical response of the film is determined by the total number of atoms that diffused into the film: $\int_{0}^{l}\varepsilon^{\prime \prime }\left(x,t\right)dx\;\sim \;\int_{0}^{l}n\left(x,t\right)dx=\int_{0}^{t}{\frac{dn\left(x,t\right)}{dx}|}_{x=l}dt$. Let us examine the dynamics of the optical response over time. Note that at long times, the concentration tends to a stationary value: $\int_{0}^{l}n\left(x,t\right)dx\to n_{0}l.$ The value of $n_{0}l$ has a sense of the specific number of atoms per unit area that penetrated the film over an infinite period of time. The difference in the specific number of particles at the time t from the stationary one $\delta n=n_{0}l-\int_{0}^{l}n\left(x,t\right)dx$ decreases exponentially.

One can find the dependence of the specific number of atoms on time at long times:

(4) $$\delta n=n_{0}l-\int_{0}^{l}n\left(x,t\right)dx\approx n_{0}l-\int_{0}^{l}\left(1-\frac{4}{\pi }\right)\cos\left(\frac{2\pi x}{l}\right)\exp\left(-\frac{t\pi^{2}D}{4l^{2}}\right)dx=\frac{8e^{-\frac{D\pi^{2}t}{4l^{2}}}l}{\pi^{2}}.$$

The time dependence found in this way approximates well the exact solution at large times. In addition, having found the characteristic response time of the system, one can find the diffusion coefficient of color centers in the WO_3_ layer:

(5) $$D=\frac{4l^{2}}{\pi^{2}t_{1}},$$

where *t*_1_ is the response time of the system, which can be found by approximating the experimental data by the $\delta n\sim e^{-t/t_{1}}$ law. Based on the calculated sensor response times, the diffusion coefficient of color centers in tungsten trioxide was found to be $D\;=\;8\;\times \;10^{-14}\;{\mathrm{cm}}^{2}s^{-1}$. That was in a good agreement with Ref. [40].

In the end, let us indicate a few promising directions for the further development of optical thin-film gas sensors. (a) Non-absorbing layered structures (photonic crystals) can be used to increase the sensitivity. Preliminary estimates show that the response amplitude of the sensing element can be increased by at least 1.5 times. These results will be reported elsewhere. (b) It is possible to increase the number of wavelengths at which measurements are made (see [41]) and for which it is possible to determine *ε*’(*t*) and *ε*”(*t*). It can be achieved via increasing the optical thickness of the dielectric layer as well as by using the waveguide modes for TE- (*s*-) polarized waves. To increase the optical thickness, one can use a protecting layer of polymer. The polymer layer can also be used to increase the selectivity of the sensor response.

## 6. Conclusions

Thus, we propose a method for measurement of the dielectric permittivity dynamics of gasochromic dielectrics interacting with gas as well as for estimating the diffusion coefficient in a layer of a gasochromic oxide. A thin-film optical gas sensor based on tungsten trioxide operating at room temperature and atmospheric pressure was demonstrated. The detection of as small as 40 ppm H_2_ in an oxygen-free atmosphere was shown.

Our calculations based on the experimental transmittance data show that a direct retrieval of the sensor response to hydrogen at a given wavelength gives an incorrect result. Particularly, relaxation times were found different. This defect was caused by complex spectral dependence of the transmittance. Correct analysis requires retrieval of the material permittivity. For this purpose, the angular dependences of the reflection coefficient were measured in dynamics for a laser wavelength of 705 nm at hydrogen concentrations in the range from 0.004% to 0.36%. The time dependence of the dielectric permittivity of tungsten trioxide interacting with hydrogen was retrieved. The errors of fitting the real and imaginary parts of dielectric permittivity of WO_3_ appeared to differ significantly; namely, the imaginary part was determined with better precision.

## Data Availability

The data presented in this study are available on request from the corresponding author.
